# What determines investment in the Nippon Individual Savings Account? an investigation of Japan’s tax-exempt investment account

**DOI:** 10.1371/journal.pone.0313433

**Published:** 2025-02-06

**Authors:** Takuya Katauke, Mostafa Saidur Rahim Khan, Yoshihiko Kadoya

**Affiliations:** School of Economics, Hiroshima University, Higashihiroshima, Japan; University of Jeddah, SAUDI ARABIA

## Abstract

In 2014, the Japanese government introduced the Nippon Individual Savings Account (NISA) to encourage tax-exempt investment and asset accumulation. In January 2024, the NISA underwent significant restructuring to boost household savings in financial asset investments. Taking advantage of the launch of the revamped NISA, we analyzed data collected in late 2022 of 95,632 active investors in a leading securities company to explore their preferences in the previous NISA. Notably, investors exhibit a preference for installment NISA over ordinary NISA, indicating an inclination for long-term capital establishment through gradual investments in secure financial assets. Regression analysis reveals that installment NISA investors are typically female, older, married, or divorced, have lower educational qualifications, engage in part-time employment, have higher incomes, display risk aversion, maintain larger asset balances, and adopt long-term perspectives. By contrast, those favoring ordinary NISA tend to be male, young, married, and financially literate and to have lower incomes, higher asset balances, and shorter-term perspectives. This study advocates maintaining both NISA types, increasing the annual and total investment limits, and eliminating tax exemption periods. These measures would be advantageous for tax savings and capital formation. However, a robust financial education campaign is emphasized to encourage individuals to invest in financial assets, breaking the tradition of keeping funds idle.

## 1. Introduction

The Japanese government introduced the Nippon Individual Savings Account (NISA), drawing inspiration from the UK’s Individual Savings Account and the US’s 401K or individual retirement account. The primary objective of the NISA is to promote tax-efficient long-term savings, encourage investment diversity (e.g., cash, stocks, bonds, and mutual funds), facilitate retirement savings, and mobilize cash reserves for investment in the stock market. Initially, the NISA comprised two variants, ordinary (*ippan*) and installment (*tsumitate*) NISA, distinguished by tax-deductible amounts and terms. Ordinary NISA, launched in 2014, offers an annual tax exemption limit of 1.2 million yen over 5 years. Installment NISA, introduced in 2018, targets smaller, longer-term investments and provides an annual tax exemption of 400,000 yen over 20 years. Despite the government’s aim to attract idle savings into investment, the data indicate that only approximately 18% of Japan’s population has invested in the NISA [1]. The plan to revamp the NISA was designed to attract more investment in financial assets [2]. In January 2024, the government announced a major reform of the NISA, expanding the annual investment limit, total allowable investment, tax exemption limit, and investor eligibility. Under the new NISA, investments can be made in either the installment or the growth framework, which is essentially similar to the previously available installments and the general NISA in terms of included securities. The major revision is in the allowable investment amount and the removal of the tax-exemption period. The installment NISA allows investment in mutual funds with an annual limit of 1.2 million yen, while the growth NISA allows investment in both stocks and mutual funds with an annual limit of 2.4 million yen. Additionally, the total investment limit has been increased to 18 million yen, and the upper ceiling of the tax exemption period has been eliminated. As the government has massively reformed the NISA, questions arise regarding why the previous NISA was not as successful, and whether the new NISA will attract investment satisfactorily.

Mobilizing idle cash and encouraging savings for investment in the financial market have been a persistent challenge for Japan. Historically, Japanese households have shown a lower inclination to invest in financial securities than Western households have [3]. Additionally, risk aversion and preference for postal savings among Japanese households contribute to general apathy toward financial market investments. In response, the government’s initiative to attract more investment in financial securities through tax exemption benefits appears promising and feasible [4]. Recent statistics from 2022 show that Japanese households currently hold more than half of their 2,000 trillion yen in financial assets as cash and savings accounts [4,5]. Despite this substantial savings, actual investment in financial securities remains low in Japan compared to the United States and Britain where households invest more of their assets in stocks and investment trusts [5–7]. Despite government efforts, the level of participation in the NISA program has been unsatisfactory [8]. A recent JITA survey revealed that 48% of individuals are aware only of the name of NISA without knowing its details, and approximately 18% of individuals have a NISA account [1,8]. The survey further indicates that NISA assets amount to 8.3 trillion yen, mainly invested in mutual funds. Consequently, compared with the targeted funds in cash and savings, participation in NISA remains notably low [9]. Recent trends clearly suggest that the government has not been successful in attracting investment in financial markets through the NISA initiative.

The provision of tax advantages through investment has been successful in many Western and developed countries in channeling household funds into higher-return-generating financial market securities. Indeed, Adam et al. [10] found that tax-exempted investment opportunities contributed to increased capital formation and stimulated economic growth by encouraging higher levels of household investment. At the household level, Choi et al. [11,12] and Crossley et al. [13] found that provisions for tax-exempted investment opportunities significantly increased household investment, particularly among middle-income households. However, Crawford and Freedman [14] found that tax-exempted investment opportunities benefited high-income households more due to their greater capacity to save and invest. Besides increasing investment, tax-exempted investment opportunities tend to increase the diversification of household investment [15] and attract risk-averse investors to financial markets [16]. The policy has long-term implications for developing retirement funds, which many people are reluctant to save for. Poterba et al. [17]and Madrian and Shea [18] studied the behavior of participants in tax-exempted investment accounts and found that such participation significantly increased retirement savings. At the national level, Engelhardt and Kumar [19] investigated the economic effects of tax-exempted investment plans and found that such plans play a significant role in the retirement landscape, potentially reducing the future burden on public pension systems. However, compared to its Western developed counterparts, the Japanese government has been less motivated by the tax-advantaged investment program, prompting the government to revamp the entire program to better address household needs.

Analyzing the preference for household investment in the financial market through the lens of demographic and socioeconomic characteristics offers valuable insights into the factors influencing individuals’ decisions to invest in financial market securities or refrain from doing so [20]. Demographic and socioeconomic factors determine the lifecycle of investment, where people’s preferences for various types of securities are influenced by different stages of life. The lifecycle of investors, as reflected in their age, plays an important role in investment decisions [18,21]. For example, Duflo and Saez [21] found that younger individuals are less likely to participate due to shorter investment horizons and competing financial priorities, while participation increases with age as individuals focus more on retirement planning. Moreover, Madrian and Shea [18] found that older employees are more likely to participate in tax-exempted investment plans, particularly when they are closer to retirement and more concerned about their financial future. However, from the viewpoint of risk-taking investment behavior, Campbell [20] found that unmarried male investors prefer to invest more in risky financial securities than older and married investors. Apart from age and gender, several studies have explored how other demographic and socioeconomic characteristics influence the decision to participate in financial markets, including tax-exempted investment accounts [10,22]. These studies consistently reveal that individuals who are male, young, unmarried, educated, financially literate, and wealthy are more likely to invest in financial market securities. Beyond demographic and socioeconomic factors, Duflo and Saez [21] and Beshears et al. [23] argued that behavioral issues such as peer effects and informational nudges play an important role in the participation in tax-exempted investment accounts. Moreover, Shoven and Sialm [24] and Poterba et al. [17] found that tax considerations play a crucial role in households’ decisions to invest in stocks. Consequently, households make asset allocation decisions based on the tax benefits derived from their investments. Vissing-Jørgensen [25] attributed the lack of investment in stocks to fixed participation costs, implying that individuals who are risk-tolerant and less liquidity-constrained are more likely to own stocks, since they can offset fixed participation costs by investing more.

In response to the insufficient investment in NISA, the government conducted a substantial restructuring in 2024 to attract more investment in financial market securities. However, there has been a notable absence of studies exploring the reasons for the lack of success of the previous NISA format. To address this gap, our study investigated the factors that motivated or discouraged investors from participating in the previous version of NISA. This study is crucial to understand whether the reform of NISA will effectively draw more investors into the financial market. Moreover, our study will help to identify whether the preferences and obstacles faced by specific demographic groups in the previous program have been addressed, thereby motivating further investment. For example, it will determine whether the new version of installment NISA has provided additional facilities for male, young, married, and financially literate individuals who did not invest much in installment NISA in the previous program. Based on previous studies on household investment preferences, we hypothesize that the demand for ordinary and installment NISA is determined by investors’ demographic and socioeconomic characteristics. Potential investors of savings-type installment NISA are likely to be female, older, married, or divorced, have lower educational qualifications, have part-time employment, show risk aversion, and adopt long-term perspectives. By contrast, those who favor ordinary NISA tend to be male, young, married, financially literate, have higher asset balances, and shorter-term perspectives. The difference in investment preferences depends on their life cycle, family responsibilities, risk-taking ability, and need for retirement savings [18,21]. Our study contributes to the existing literature in at least two ways. First, it provides explanations for the success and failure of participation in NISA by Japanese investors. Second, it offers insights into retirement savings and why some individuals lag behind in accumulating sufficient funds for their retirement.

## 2. Data and methods

### 2.1. Data

The study was designed as a cross-sectional survey. The data collection was carried out through an online survey conducted by Rakuten Securities and Hiroshima University. This survey was designed to capture a snapshot of the demographic, socioeconomic, and personality characteristics and preferences of Japanese adults at the end of 2022. The selection criteria for participation in the survey required participants to be active account holders of Rakuten Securities, aged 18 years or older, and willing and able to complete the online questionnaire. The active account holders mean those who have logged in to the security account at least once over the last year and have purchased at least one item before. The survey was conducted online among active account holders of Rakuten Securities aged 18 years and older. The data collection period lasted for two months, from November to December 2022, and included questions on the demographic, socioeconomic, and personal preference and characteristics of Japanese adults. The sampling strategy was convenience-based, as it relied on the existing customer base of Rakuten Securities. All participants who met the selection criteria were invited to complete the online questionnaire. This approach ensured that the survey included respondents from various socioeconomic backgrounds, given the diverse clientele of the securities company. However, the reliance on voluntary participation among the company’s active account holders introduces a potential selection bias, as the sample may not fully represent the general population of Japanese adults. Nonetheless, the large sample size and breadth of the questions covered help mitigate some of these limitations, providing a substantial dataset for analysis. After excluding missing data, the final sample size comprised 95,632 observations, representing 73.56% of valid responses. This exclusion could have potentially affected the overall representativeness of the sample. However, a comparison of the data distribution before and after removing observations with missing values revealed that there were no significant differences that could materially affect the results. Therefore, the final dataset appears to be sufficient and representative, capable of yielding unbiased results.

### 2.2. Ethics statement

Review and approval by an ethics committee was not needed for this study because this study does not involve animal experiments, human and behavior studies. However, the authors obtained written informed consent from the participants to partake in the survey.

### 2.3. Variables

The dependent variable is NISA investment. The dataset contains information on the NISA account-opening status. Customers who had opened a NISA account were coded as 1, and otherwise 0. To examine the respondents’ NISA status in more detail, we divided them into two types: ordinary NISA (*ippan NISA*) and installment NISA (*tsumitate NISA*). Ordinary_NISA is a binary variable coded 1 if the customer had opened an ordinary NISA account and Installment_NISA is a binary variable coded 1 if the customer had opened an installment NISA account. Under the NISA system, citizens can only open an ordinary or installment NISA. No overlap is allowed between the two types of NISA.

The explanatory variables include investors’ demographic, socioeconomic, and personality characteristics, such as sex, age, marital status, years of education, household income, household assets, and unemployment. Moreover, we included financial literacy as a measure of the respondents’ financial market knowledge. In our study, financial literacy plays an important role, because financially literate people are more likely to invest in NISA because of their knowledge, trust, and ability to reduce the cost of participation in the financial market. Previous studies also find that financial literacy plays a significant role in stock market participation [6,7,26]. To measure financial literacy, we adopted the well-utilized methodology of Lusardi and Mitchell [27–30]). Based on the answers to the three questions (see S1 Appendix), we measured the level of customers’ financial literacy. We assigned 1 point for each correct answer and 0 for each incorrect answer. We then created a financial literacy index by calculating the average scores. Furthermore, we controlled for risk aversion and a myopic view of the future. The detailed definitions of the variables are presented in Table 1.

**Table 1:** Definitions and measurement of variables.

Variable	Definition
NISA	A binary indicator for NISA investment (ordinary NISA + installment NISA), which equals 1 if respondents have a NISA account, and 0 otherwise.
Ordinary_NISA (Ippan NISA)	A binary indicator for ordinary NISA investment, which equals 1 if respondents have an ordinary NISA account, and 0 otherwise.
Installment_NISA (Tsumitate NISA)	A binary indicator for installment NISA investment, which equals 1 if respondents have an installment NISA account, and 0 otherwise.
Financial Literacy	Continuous variable: average score for the number of correct answers to the three financial literacy questions.
Sex	Binary variable: 1 = male, 0 = female
Age	Age of participants
Years of education	Continuous variable: respondents’ years of education
Married	Binary variable: 1 = married, 0 = otherwise
Divorce	Binary variable: 1 = divorced, 0 = otherwise
Household income	Continuous variable: Annual earned income before taxes and with bonuses of the entire household in 2022 (unit: million yen)
Household assets	Continuous variable: Balanced amount of financial assets (savings, stocks, insurance, etc.) of the entire household (unit: million yen)
Unemployed	Binary variable: 1 = those who have no current job, 0 = otherwise
Risk aversion	Continuous variable: risk of rain preference (percentage score from the question, “Usually when you go out, how high must the probability of rainfall be before you take an umbrella?”)
Myopic view of the future	5-scale measure: respondents’ perceptions about the future, measured based on the answer to this item: “Since the future is uncertain, it is a waste to think about it” (1 = completely disagree, 5 = completely agree)
Instrumental variable	Definition
Language_ability	Instrumental variable of financial literacy: the 5-scale degree of Japanese language ability in high school based on the answer to the following question: “Regarding your Japanese language performance in high school, please select the answer that applies to you from the list below” (1 = very bad, 5 = very good).

### 2.4. Statistical analysis and procedures

The regression models used in this study investigated the role of sociodemographic factors in investment decision-making between ordinary and installment NISA accounts. We employed three regression models for this purpose, with overall investment in NISA, investment in ordinary NISA, and investment in installment NISA as the dependent variables, respectively, while keeping the independent variables consistent across models. Given the differences between ordinary and installment NISA, such as investment amount, timeframe, and selection of securities, the second and third models illustrate how Japanese households value these distinctions. STATA software was used to conduct the regression analysis. The regression models are as follows [6]:

NISA(1=haveaNISA,0=otherwise)

$$=\beta_{0}+\beta_{1}{Financial\;Literacy}_{i}(IV:{language\_ability}_{i})+\beta_{2}{sex}_{i}+\beta_{3}{age}_{i}+\beta_{4}{married}_{i}+\beta_{5}{divorced}_{i}+\beta_{6}{years\;of\;education}_{i}+\beta_{7}{household\;income}_{i}+\beta_{8}{household\;assets}_{i}+\beta_{9}{fulltime}_{i}+\beta_{10}{risk\_aversion}_{i}+\beta_{11}{myopics\;view\;of\;the\;future}_{i}+\varepsilon_{i}$$ [1]

IPPAN_NISA(1=haveanordinaryNISA,0=otherwise)

$$=\beta_{0}+\beta_{1}{Financial\;Literacy}_{i}(IV:{language\_ability}_{i})+\beta_{2}{sex}_{i}+\beta_{3}{age}_{i}+\beta_{4}{married}_{i}+\beta_{5}{divorced}_{i}+\beta_{6}{years\;of\;education}_{i}+\beta_{7}{household\;income}_{i}+\beta_{8}{household\;assets}_{i}+\beta_{9}{fulltime}_{i}+\beta_{10}{risk\_aversion}_{i}+\beta_{11}{myopics\;view\;of\;the\;future}_{i}+\varepsilon_{i}$$ [2]

TSUMITATE_NISA(1=haveaninstallmentNISA,0=otherwise)

$$=\beta_{0}+\beta_{1}{Financial\;Literacy}_{i}(IV:{language\_ability}_{i})+\beta_{2}{sex}_{i}+\beta_{3}{age}_{i}+\beta_{4}{married}_{i}+\beta_{5}{divorced}_{i}+\beta_{6}{years\;of\;education}_{i}+\beta_{7}{household\;income}_{i}+\beta_{8}{household\;assets}_{i}+\beta_{9}{fulltime}_{i}+\beta_{10}{risk\_aversion}_{i}+\beta_{11}{myopics\;view\;of\;the\;future}_{i}+\varepsilon_{i}$$ [3]

where FL represents the average financial literacy score, X is a vector of individual characteristics, and ***ε***_***i***_ indicates the error term. Because the dependent variables in each specification are binary, we employed a probit model to estimate the equations.

However, previous studies have argued that there is a potential endogeneity concern between financial literacy and investment behavior (e.g., [31–33]). Furthermore, there may be reverse causality between financial literacy and asset accumulation. Although we investigated the association between financial literacy and NISA investment, the desire to accumulate more wealth could be an incentive to invest in financial knowledge, which could produce endogeneity and biased estimation results [31]. Furthermore, the omitted variables can affect both financial literacy and NISA investments. It is possible that an early endowment influences current levels of financial literacy [32,33]. For instance, Herd et al. [34] showed that the current level of financial literacy is strongly associated with cognitive ability at a young age.

To reduce these biases, we applied the instrumental variable (IV) method for our analysis. We used the level of Japanese language ability in high school as the IV, which shows the respondents’ cognitive ability before entering the labor market. Thus, we can mitigate the bias of omitted variables caused by early endowment. Moreover, as Sekita [35] argued, respondents must be able to comprehend sentences in financial literacy questions they are required to answer, whereas Japanese language skills are not directly linked to investment behavior. Thus, following the literature [35,36], we used Language_ability, measured by language skills in high school, as the IV in all equations. Respondents were asked, “Regarding your Japanese language performance in high school, please select the answer that applies to you from the list below,” with five possible responses (1 = very bad, 5 = very good).

Following Sekita [35] and Thomas and Spataro [33], we tested the validity of the instruments. First, we used Wald’s test for exogeneity. The results reject the null hypothesis that financial literacy is an exogenous variable, particularly for models in which the dependent variables are NISA and ordinary NISA investments. In addition, the Durbin–Wu–Hausman test results show a possible concern regarding the endogeneity between NISA investment, ordinary NISA investment, and financial literacy. We also tested the validity of Language_ability as the IV. The first-stage regression results show that the instrument is significantly correlated with financial literacy, and the F-statistics significantly exceed the 10% critical value. Thus, our IV, Language_ability, is not weak. The test results are not reported to save space but are available upon request.

To assess the presence of multicollinearity in the models, we conducted a variance inflation factor (VIF) test. The results of the VIF test indicated that there was no evidence of multicollinearity among the model variables (the results are available upon request).

## 3. Empirical results

### 3.1. Descriptive statistics

Table 2 presents the descriptive statistics of the sample. The results show that approximately 71.3% of the respondents in our dataset opened a NISA account. More than half of the respondents had opened an installment NISA account. By contrast, the percentage of respondents utilizing ordinary NISA was lower than that of respondents utilizing installment NISA (approximately 20%). The preference for installment NISA can be attributed to investors’ preference for gradual investments in secured financial securities.

**Table 2:** Summary statistics of the study variables.

Variable	Obs.	Mean	STDEV	Min	Max
NISA	95632	.7134118	.4521698	0	1
Ordinary_NISA	95632	.1922474	.3940684	0	1
Installment_NISA	95632	.5211645	.4995545	0	1
Financial literacy	95632	.8019457	.2916041	0	1
Early cognition	95632	3.241206	.9388287	1	5
Sex	95632	.6410302	.4797008	0	1
Age	95632	43.87233	12.21042	20	109
Married	95632	.6674021	.4711463	0	1
Divorced	95632	.0597394	.2370047	0	1
Years of education	95632	15.21264	2.005791	10.5	21
Household income	95632	7540907	4133957	1000000	2.00e+07
Log of household income	95632	15.67657	.5959455	13.81551	16.81124
Household assets	95632	1.86e+07	2.32e+07	2500000	1.00e+08
Log of household assets	95632	16.13666	1.070539	14.7318	18.42068
Unemployed	95632	.0547306	.2274549	0	1
Risk aversion	95632	.5397953	.2293759	0	1
Myopic view of the future	95632	2.325717	.9521124	1	5

Regarding sociodemographic characteristics, approximately 64.1% of the respondents were male, with an average age of 43.9 years. Approximately 66.7% of the respondents were married and 6.0% were divorced. On average, the respondents had 15.2 years of schooling, approximately 7.54 million yen in annual household income, and 18.6 million yen in household financial assets. By contrast, 5.5% of the respondents were unemployed. The results further show that the respondents’ average financial literacy level was 0.80. Furthermore, respondents rated their Japanese language ability in high school (a proxy for early cognitive skills) as 3.24 out of 5 on average. Regarding personality factors, the respondents assessed their level of risk aversion as approximately 0.54, and their average level of myopic view of the future as 2.33 on a scale of 5.

### 3.2. Regression results

Table 3 presents the estimation results, using NISA investment as the dependent variable. We report both the probit and IV probit regression results to demonstrate the impact of endogeneity. In both probit and IV probit regressions, we present the results of the three specifications differentiated by the control variables. The probit regression results show that financial literacy is negatively associated with NISA investment at the 1% significance level. However, as discussed in the previous section, there is potential concern about the endogeneity between NISA investments and financial literacy, which is supported by several statistical tests. Therefore, we present our results by using an IV probit regression model. Contrary to the results before using the IV, financial literacy does not show a significant relationship with NISA investment after controlling for sociodemographic factors. For the other explanatory variables, male sex, age, years of education, and household assets are negatively related to NISA investment in all models. By contrast, being married, divorced, or unemployed has a significantly positive relationship.

**Table 3:** Probit and IV probit estimations for NISA.

Variable	Probit			IV Probit		
	Model 1.1	Model 1.2	Model 1.3	Model 1.4	Model 1.5	Model 1.6
financial_literacy	-0.2313***	-0.1387***	-0.1439***	-0.3480**	0.3436	0.3557
	(0.0161)	(0.0164)	(0.0165)	(0.1454)	(0.2162)	(0.2333)
Sex	-0.2632***	-0.2618***	-0.2630***	-0.2487***	-0.3111***	-0.3118***
	(0.0099)	(0.0100)	(0.0100)	(0.0205)	(0.0231)	(0.0236)
Age	-0.0269***	-0.0237***	-0.0236***	-0.0268***	-0.0236***	-0.0235***
	(0.0004)	(0.0005)	(0.0005)	(0.0004)	(0.0005)	(0.0005)
Married	0.0999***	0.1043***	0.1026***	0.1007***	0.1123***	0.1120***
	(0.0106)	(0.0116)	(0.0116)	(0.0106)	(0.0120)	(0.0122)
Divorced	0.1096***	0.0687***	0.0669***	0.1051***	0.0776***	0.0771***
	(0.0204)	(0.0205)	(0.0205)	(0.0212)	(0.0208)	(0.0210)
years_of_educ		-0.0157***	-0.0157***		-0.0239***	-0.0238***
		(0.0024)	(0.0024)		(0.0043)	(0.0044)
log_of_hincome		0.0272***	0.0269***		0.0117	0.0114
		(0.0096)	(0.0096)		(0.0119)	(0.0120)
log_of_hasset		-0.1200***	-0.1206***		-0.1375***	-0.1374***
		(0.0051)	(0.0051)		(0.0089)	(0.0088)
Fulltime		0.0420*	0.0423**		0.0494**	0.0498**
		(0.0214)	(0.0214)		(0.0215)	(0.0215)
risk_aversion			-0.0363*			-0.0320
			(0.0198)			(0.0198)
Myopic view of the future			-0.0139***			0.0021
			(0.0048)			(0.0088)
Constant	2.0637***	3.5985***	3.6654***	2.1424***	3.8772***	3.8778***
	(0.0209)	(0.1332)	(0.1350)	(0.0985)	(0.1724)	(0.1578)
Observations	95,632	95,632	95,632	95,632	95,632	95,632
Log likelihood	-53637	-53247	-53242	-68611	-65929	-65400
Chi2 statistics	7095	7817	7826	6971	7863	7878
p-value	0	0	0	0	0	0
Wald test				0.650	4.910**	4.515**

Note: Robust standard errors are shown in parentheses.

*** p<0.01

** p<0.05

* p<0.1.

Table 4 presents the regression results for the factors associated with ordinary NISA investments. Our results show that financial literacy is positively associated with ordinary NISA investments at the 1% level, even after dealing with endogeneity issues. Moreover, being male, age, household assets, being married, and having a myopic view of the future have a positive association with ordinary NISA investment, whereas household income has a negative association.

**Table 4:** Probit and IV probit estimations for ordinary NISA.

Variable	Probit			IV Probit		
	Model 2.1	Model 2.2	Model 2.3	Model 2.4	Model 2.5	Model 2.6
financial_literacy	0.1422***	0.0804***	0.0818***	0.7077***	0.5010**	0.5438**
	(0.0173)	(0.0178)	(0.0179)	(0.1521)	(0.2338)	(0.2517)
Sex	0.1690***	0.1697***	0.1701***	0.0976***	0.1234***	0.1211***
	(0.0107)	(0.0108)	(0.0108)	(0.0225)	(0.0285)	(0.0295)
Age	0.0236***	0.0207***	0.0207***	0.0228***	0.0204***	0.0204***
	(0.0004)	(0.0005)	(0.0005)	(0.0005)	(0.0005)	(0.0006)
Married	0.0202*	0.0163	0.0167	0.0159	0.0242*	0.0262*
	(0.0116)	(0.0128)	(0.0128)	(0.0116)	(0.0134)	(0.0137)
Divorced	-0.0307	-0.0005	-0.0001	-0.0083	0.0080	0.0100
	(0.0220)	(0.0222)	(0.0222)	(0.0227)	(0.0226)	(0.0228)
years_of_educ		0.0062**	0.0062**		-0.0012	-0.0015
		(0.0025)	(0.0025)		(0.0048)	(0.0049)
log_of_hincome		-0.0100	-0.0099		-0.0233*	-0.0240*
		(0.0103)	(0.0103)		(0.0126)	(0.0128)
log_of_hasset		0.0867***	0.0869***		0.0699***	0.0695***
		(0.0054)	(0.0054)		(0.0110)	(0.0112)
Fulltime		0.0254	0.0253		0.0320	0.0323
		(0.0221)	(0.0221)		(0.0224)	(0.0224)
risk_aversion			0.0064			0.0100
			(0.0214)			(0.0214)
Myopic view of the future			0.0039			0.0186**
			(0.0052)			(0.0095)
Constant	-2.1822***	-3.3460***	-3.3646***	-2.5410***	-3.0503***	-3.1071***
	(0.0228)	(0.1430)	(0.1449)	(0.0911)	(0.2271)	(0.2119)
Observations	95,632	95,632	95,632	95,632	95,632	95,632
Log likelihood	-44470	-44302	-44301	-59437	-56984	-56460
Chi2 statistics	4602	5008	5009	4806	5131	5154
p-value	0	0	0	0	0	0
Wald test				13.41***	3.186**	3.301**

Note: Robust standard errors are shown in parentheses.

*** p<0.01

** p<0.05

* p<0.1.

Table 5 presents the regression results for the factors associated with NISA investments. These results indicate that financial literacy negatively affects participation in NISA installments in the first specification. When we deal with the potential endogeneity bias using the IV probit model, financial literacy does not have a significant relationship with NISA investments. Regarding other explanatory variables, being male, age, years of education, household assets, unemployment, risk aversion, and a myopic view of the future are negatively associated with installment NISA investments, whereas being married, being divorced, and household income have a positive association.

**Table 5:** Probit and IV probit estimations for installment NISA.

Variable	Probit			IV Probit		
	Model 3.1	Model 3.2	Model 3.3	Model 3.4	Model 3.5	Model 3.6
financial_literacy	-0.2780***	-0.1466***	-0.1511***	-0.9169***	-0.2029	-0.2257
	(0.0153)	(0.0157)	(0.0158)	(0.1336)	(0.2145)	(0.2317)
Sex	-0.3452***	-0.3453***	-0.3461***	-0.2607***	-0.3392***	-0.3383***
	(0.0093)	(0.0095)	(0.0095)	(0.0211)	(0.0251)	(0.0261)
Age	-0.0449***	-0.0387***	-0.0386***	-0.0436***	-0.0387***	-0.0386***
	(0.0004)	(0.0005)	(0.0005)	(0.0006)	(0.0005)	(0.0005)
Married	0.0993***	0.0813***	0.0795***	0.1021***	0.0802***	0.0779***
	(0.0102)	(0.0114)	(0.0114)	(0.0102)	(0.0121)	(0.0124)
Divorced	0.1857***	0.1059***	0.1039***	0.1577***	0.1047***	0.1023***
	(0.0200)	(0.0202)	(0.0202)	(0.0209)	(0.0206)	(0.0208)
years_of_educ		-0.0254***	-0.0252***		-0.0244***	-0.0240***
		(0.0023)	(0.0023)		(0.0044)	(0.0045)
log_of_hincome		0.0631***	0.0629***		0.0649***	0.0652***
		(0.0096)	(0.0096)		(0.0117)	(0.0119)
log_of_hasset		-0.1893***	-0.1895***		-0.1872***	-0.1868***
		(0.0050)	(0.0050)		(0.0098)	(0.0099)
Fulltime		-0.2513***	-0.2507***		-0.2522***	-0.2519***
		(0.0253)	(0.0253)		(0.0256)	(0.0256)
risk_aversion			-0.0478**			-0.0483**
			(0.0193)			(0.0194)
Myopic view of the future			-0.0119**			-0.0143*
			(0.0047)			(0.0087)
Constant	2.3890***	4.4880***	4.5453***	2.7889***	4.4512***	4.5074***
	(0.0209)	(0.1318)	(0.1335)	(0.0779)	(0.1945)	(0.1804)
Observations	95,632	95,632	95,632	95,632	95,632	95,632
Log likelihood	-57003	-55981	-55975	-71966	-68665	-68135
Chi2 statistics	16697	17473	17483	17595	17433	17453
p-value	0	0	0	0	0	0
Wald test for endogeneity				21.77***	0.0692	0.104

Note: Robust standard errors are shown in parentheses.

*** p<0.01

** p<0.05

* p<0.1.

## 4. Discussion

In 2014, the Japanese government introduced the NISA as a strategy to redirect a significant amount of idle cash toward long-term capital formation. Faced with a lack of interest among Japanese households, the government substantially revised the NISA in January 2024 to enhance its appeal and attract more investment in financial markets. This study aimed to identify the factors that are positively and negatively associated with investments in the original NISA, providing insights into whether the revised NISA would attract prospective investors. The preliminary uncontrolled descriptive statistics indicate that 71% of investors embraced NISA, with 19% opting for the ordinary variant and 52% for the installment variant. The preference for installment NISA, which facilitates investment in mutual funds, suggests that households are primarily interested in gradually accumulating long-term secure capital. In the revamped NISA, the government increased the annual allowable investment limit and expanded the total lifetime investment limit for installment NISA while eliminating the tax exemption period. These revisions are considered favorable to investors and are expected to encourage further investment in installment NISA. By comparison, investment in general NISA (now equivalent to growth NISA), which involves investing in stocks, is less attractive in Japan. This can be attributed to the higher risks associated with the scheme and the absence of tax benefits to reduce stock prices. Despite the government’s efforts to increase the investment limit and abolish the tax exemption period, the potential losses from active stock investments have not been adequately addressed. Therefore, the success of growth NISA is believed to hinge on market conditions; it may attract sufficient funds in a growing stock market scenario.

We analyze NISA participation using a sociodemographic characterization framework, wherein investors make portfolio choices based on their demographic and socioeconomic backgrounds and preferences for risk and return [20,36,37]. Our estimation results reveal a significantly positive association between financial literacy and ordinary NISA. In this variant, investors are eligible to invest in various financial securities including stocks, exchange-traded funds, real estate investment trusts, and mutual funds. This finding aligns with previous studies suggesting that financial literacy enhances people’s understanding of active investment and reduces the cost of participation in the financial market [6,7,31,33,38,39]. However, the association between financial literacy and installment NISA was not significant after controlling for endogeneity. We posit that, in contrast to investment knowledge, investing in installment NISA requires savings motivation and a future-oriented perspective.

In our exploration of financial literacy’s impact on ordinary NISA investments, we acknowledge the significant role played by the range of securities available in the investment basket. Ordinary NISA offers a broader array of financial securities than installment NISA, potentially enhancing its appeal for profit maximization and risk mitigation. Bianchi [40] highlighted that individuals with higher financial literacy tend to hold a greater proportion of risky securities with the aim of optimizing returns than those who are financially less savvy. Furthermore, substantial tax exemptions from larger investments within ordinary NISA present an effective strategy for reducing investor costs. Unlike ordinary NISA, our study found no significant association between financial literacy and installment NISA. Installment NISA seems to function more as a savings product than as a practical investment opportunity for active investors. Therefore, investors’ inclination toward gradually building long-term capital through installments demands patience rather than extensive financial knowledge. To reinforce this perspective, we note that investors who favored installment NISA typically exhibited a long-term outlook.

Among the demographic, socioeconomic, and personality variables, we observed distinct patterns between ordinary and installment NISA. On the one hand, investors likely to have an ordinary NISA have the following characteristics: male, young, married, low-income, higher balance of assets, and a myopic view of the future. These characteristics indicate that the desire for higher income, a risk-taking attitude, and a short investment timeframe signify ordinary NISA investment. These investor characteristics are consistent with the features of ordinary NISA, such as providing the opportunity to invest in risky securities, having a short timeframe, and being more of an investment product than a savings product. On the other hand, investors likely to have an installment NISA have the following characteristics: female, older, married, divorced, low education, full-time unemployment, higher income, higher balance of assets, risk aversion, and a long-term view of the future. These characteristics indicate that the desire to secure future income, a low risk-taking attitude, and a long-term investment framework signify installment NISA investment. These investor characteristics are consistent with the features of installment NISA, such as allowing investments only in mutual funds, having a long timeframe, and being more of a savings product than an investment.

Regarding the NISA reformation, our findings support the maintenance of the two types of NISA. Although investors predominantly favor installment NISA, some investors prefer direct investment in stocks and other financial securities. Furthermore, the elimination of the annual limit for investment and the expansion of the ceiling for total investment in both types of NISA would offer additional tax advantages. Removing the tax exemption period limit is also a positive aspect from the perspective of tax benefits, potentially attracting new investors to NISA. However, the current reform does not permit tax savings from the reduction in security prices, which may discourage some investors from opting for NISA. Finally, we assert that holding cash and maintaining low- or no-interest savings accounts are long-standing traditions among Japanese households. In addition to the evident benefits, authorities need to educate people, especially the older generation, about the advantages of investment and the costs associated with keeping idle money.

Our study has several limitations. First, because our data are exclusively sourced from a single securities company, the validity of our findings may be compromised if investors have opened NISA accounts with different securities firms. Second, the data collection period during the COVID-19 pandemic could have affected the choice of investment and behavioral patterns to some extent. Third, the results should be generalized with caution, because the data were collected from active investors. Finally, we had to exclude 26.44% of cases due to missing data. While our analysis indicated no significant differences in the distribution of key demographic, socioeconomic, and psychological variables before and after this exclusion, the potential for bias remains. Nevertheless, this study explained investment in NISA using a sociodemographic characterization framework and identified the factors associated with the decision to invest in NISA.

## 5. Conclusion

The Japanese government recently underwent a significant revision to the NISA, aiming to attract more individuals to accumulate assets through tax-exempt investments in financial market securities and funds. In this context, the reasons for the previous NISA’s lack of success and the potential success of the reformed NISA remain crucial questions. Using large-scale data collected in late 2022, this study explores the factors associated with NISA investments through the sociodemographic characterization framework of investors and assesses the probability of success of the revised program. Our uncontrolled observations reveal a significantly higher preference for installment NISA than ordinary NISA. The regression results indicate a positive association between financial literacy and ordinary NISA, but not with installment NISA. Additionally, investors who are likely to opt for ordinary NISA exhibit the following characteristics: male, young, married, low-income, higher asset balance, and a myopic view of the future. Meanwhile, investors inclined toward installment NISA demonstrate the following traits: being female, older, married or divorced, lower education, full-time unemployment, higher income, increased asset balance, risk aversion, and a long-term perspective for the future.

We believe that the withdrawal of the annual investment limit and expansion of the ceiling for total investment in both types of NISA offer additional tax advantages. Additionally, the removal of the tax exemption period limit is viewed positively in terms of tax benefits and is expected to attract new investors to the NISA. However, the current reform does not allow tax savings from a reduction in security prices, a factor that could dissuade some investors from choosing a NISA account. Finally, we maintain that holding cash and maintaining low- or no-interest savings accounts are long-standing traditions in Japanese households. Our findings suggest that, considering the established practice of holding cash, authorities should place greater emphasis on educating people, particularly the older generation, about the benefits of investment and the costs associated with keeping idle money.

## Supporting information

S1 Appendix (DOCX)

S1 Data (XLSX)
