# Supplementary material for: What determines investment in the Nippon Individual Savings Account? an investigation of Japan’s tax-exempt investment account
Source: PLoS One. 2025 Feb 6;20(2):e0313433. doi: 10.1371/journal.pone.0313433 (PMC11801543; doi:10.1371/journal.pone.0313433)
Supplement: S1 Appendix — (DOCX) [file pone.0313433.s001.docx]

**Appendix A**

Q13. Assume that you have 10,000 yen in your savings account and the interest rate is 2% per year. Furthermore, we assume that the deposited money and interest are never withdrawn from the account. Five years later, how much savings would you receive? Please choose one of the following four options.

1_ Over 10,200 yen

2_ Exactly 10,200 yen

3_ Less than 10,200 yen

4_ I don’t know

Q14. Suppose the interest rate on your savings account is 1% per year and the inflation rate is 2% per year. After 1 year, how much money do you think you can have from that account?

1_ I can buy more things than today

2_ I can buy the same number of things as today

3_ I can buy less than I can today

4_ I don’t know

Q15. Tell us what you think is closest to the following sentence: Buying the stock of a single company is generally a safer investment than buying stock investment trust.

1_ True

2_ False

3_ I don’t know
